# Shear Modulus Estimation on *Vastus Intermedius* of Elderly and Young Females over the Entire Range of Isometric Contraction

**DOI:** 10.1371/journal.pone.0101769

**Published:** 2014-07-03

**Authors:** Cong-Zhi Wang, Tian-Jie Li, Yong-Ping Zheng

**Affiliations:** 1 Paul C. Lauterbur Research Center for Biomedical Imaging, Institute of Biomedical and Health Engineering, Shenzhen Institutes of Advanced Technology, Chinese Academy of Sciences, Shenzhen, China; 2 Interdisciplinary Division of Biomedical Engineering, the Hong Kong Polytechnic University, Hong Kong, China; 3 Beijing Center for Mathematics and Information Interdisciplinary Sciences, Beijing, China; Semmelweis University, Hungary

## Abstract

Elderly people often suffer from sarcopenia in their lower extremities, which gives rise to the increased susceptibility of fall. Comparing the mechanical properties of the knee extensor/flexors on elderly and young subjects is helpful in understanding the underlying mechanisms of the muscle aging process. However, although the stiffness of skeletal muscle has been proved to be positively correlated to its non-fatiguing contraction intensity by some existing methods, this conclusion has not been verified above 50% maximum voluntary contraction (MVC) due to the limitation of their measurement range. In this study, a vibro-ultrasound system was set up to achieve a considerably larger measurement range on muscle stiffness estimation. Its feasibility was verified on self-made silicone phantoms by comparing with the mechanical indentation method. The system was then used to assess the stiffness of *vastus intermedius* (VI), one of the knee extensors, on 10 healthy elderly female subjects (56.7±4.9 yr) and 10 healthy young female subjects (27.6±5.0 yr). The VI stiffness in its action direction was confirmed to be positively correlated to the % MVC level (R^2^ = 0.999) over the entire range of isometric contraction, i.e. from 0% MVC (relaxed state) to 100% MVC. Furthermore, it was shown that there was no significant difference between the mean VI shear modulus of the elderly and young subjects in a relaxed state (p>0.1). However, when performing step isometric contraction, the VI stiffness of young female subjects was found to be larger than that of elderly participants (p<0.001), especially at the relatively higher contraction levels. The results expanded our knowledge on the mechanical property of the elderly’s skeletal muscle and its relationship with intensity of active contraction. Furthermore, the vibro-ultrasound system has a potential to become a powerful tool for investigating the elderly’s muscle diseases.

## Introduction

Sarcopenia refers to the degenerative decline of muscle strength in the elderly [1]. It will significantly increase their risk of sudden falls and dramatically impact on their quality of life [2], [3]. As the population of elderly people continues to escalate, sarcopenia carries more burdens of public health care and social services. To reveal the process and mechanism of this disease, some studies have focused on the morphological change of skeletal muscle caused by age. Muscle features including size, fascicle length and pennation angle have been extensively investigated [4]–[7]. Moreover, some other studies applied biomedicine methods on elderly patients to investigate the fiber atrophy on different fiber types [8], [9] and the phenomenon of “metabolic dysregulation”, such as impaired oxidative defense and decreased mitochondrial function [10], [11].

Besides these studies, mechanical properties, especially the stiffness of skeletal muscle, have also attracted broad research interest. Skeletal muscle stiffness has been verified to contribute significantly to action efficiency [12], [13], hence its quantification measurement in vivo can help to improve the understanding of functional changes in muscle. Several experimental techniques including “quick release” and “sinusoidal perturbations” were developed to study the global mechanical properties of musculotendinous and musculoarticular complexes, but the various components of these complexes cannot be differentiated [14], [15]. Furthermore, indentation devices, such as myotonometer [16], [17] and Tissue Ultrasound Palpation System (TUPS) [18], [19] have been developed to assess the muscle stiffness locally. Although these devices have acceptable reliability, muscle stiffness at different depths cannot be fully distinguished and the stiffness in muscle action direction cannot be obtained.

In the last two decades, elastography, the technology for noninvasively measuring or imaging the mechanical characteristics of soft tissue, has been rapidly evolving and applied in many clinical areas, such as the diagnosis of liver fibrosis and breast cancer [20]. Several quantitative elastography methods based on shear wave velocity estimation have also been proposed to quantify the elastic modulus of a single muscle. In a simple model of pure elastic, locally homogeneous and isotropic material, its shear modulus *µ* is related to the shear wave velocity *c_s_* propagating in it via the following equation:
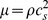
(1)where *ρ* is the mass density of the material. Although the assumptions of this model are not fully satisfied for skeletal muscle, they are generally assumed to yield an acceptable estimation of muscle stiffness and widely used by many methods [21]–[25].

In sonoelastography, tissues were stimulated with a low-frequency (10 to 1000 Hz) mechanical vibration. The induced tissue movement was measured by a Doppler instrument for obtaining the shear wave velocity [26], [27]. Using this technique, Levinson et al. reported that the shear modulus of quadriceps femoris was positively correlated to the increasing load imposed on it [28]. However, the method requires a long acquisition time. When it is used on a muscle during high intensity contraction, the muscle will soon fatigue and the results will be different from those under normal condition. In transient elastography method, an ultrasound transducer was used as a piston-like vibrator to apply a pulsed excitation on the muscle, so the shear wave propagating perpendicularly to the muscle action direction could be studied [23]. This method has been used to assess the shear modulus of biceps brachii and gastrocnemius medius in the transverse direction [29], [30]. However, since skeletal muscle is anisotropic, shear modulus in the muscle action direction is more important for muscle functional assessment. It has been reported that the shear modulus of biceps brachii in the muscle action direction was approximately 4 times larger than that in the perpendicular direction in a relaxed state and about 9 times larger during a low intensity isometric contraction [31]. Shear wave dispersion ultrasound vibrometry (SDUV) method can measure the muscle stiffness in its action direction [24]. It has been evaluated on animal muscles [32], but not on human muscle in vivo, since the results were not fully satisfactory. Acoustic radiation force induced by a focused ultrasound beam was used to generate a vibration source in the muscle and the propagation of shear wave was monitored by the detection beam. Shear wave velocity was then calculated from the time delay and distance between the two beams. This method has difficulty in generating decent waves in a deep muscle [33]. Another method named as supersonic shear imaging (SSI) has been reported to study the shear wave propagation in 2D plane [34]. After generating the shear wave within a tissue by acoustic radiation force, its propagation was monitored by a series of B-mode images at an ultra-high frame rate. SSI has been applied to study the stiffness of biceps brachii [35], lower leg muscles [36] and finger muscles [37]. However, the applied depth of SSI was also limited by the intensity of acoustic radiation force for safety consideration. In addition, it was reported that the measurement of SSI system would saturate at a shear modulus value of 266 kPa [37]. Therefore, the highest muscle contraction level in their study was only approximate 50% of maximum voluntary contraction (MVC) torque (“%MVC” is a generally used indicator for representing relative contraction intensity of muscle). Magnetic resonance elastography (MRE) is another technique which can visualize the propagation of shear wave excited by an external vibrator [25]. The wavelength can be measured from MRE images and then used to calculate shear wave velocity. In contrast to ultrasound, MRE has no limitation in penetration depth and can simultaneously provide images with high resolution. Many studies have been reported to assess muscle stiffness using MRE [38]–[41]. However, it requires very long acquisition time (about 1–3 minutes for each measurement) which greatly limits its application on skeletal muscle, particularly during a high intensity contraction. The signal-to-noise ratio (SNR) of MRE images was reported to be significantly reduced when a high intensity muscle contraction was performed [42].

Based on these methods, many previous studies have reported the positive correlation between the muscle stiffness and the non-fatiguing muscle contraction intensity. However, rare studies have been performed to quantitatively assess the muscle stiffness of the elderly in relaxed and isometric contraction conditions. Domire et al. measured the stiffness of tibialis anterior muscle on 16 elderly females (mean age: 60±7.2 yr) at a relaxed condition using MRE [43], and reported that the mean shear modulus was 10.0±2.9 kPa and there was no significant relationship between age and the relaxed muscle stiffness. To our knowledge, all the methods mentioned above cannot cover the entire range of muscle contraction, i.e. from 0% to 100% MVC, due to the limitation of their measurement range. For example, it was reported that the satisfactory quantitative results could be only achieved under 20% MVC torque level using MRE [42], while the muscle stiffness was only assessed during a contraction equal to about 50% MVC level using SSI [37].

**Figure 1:**
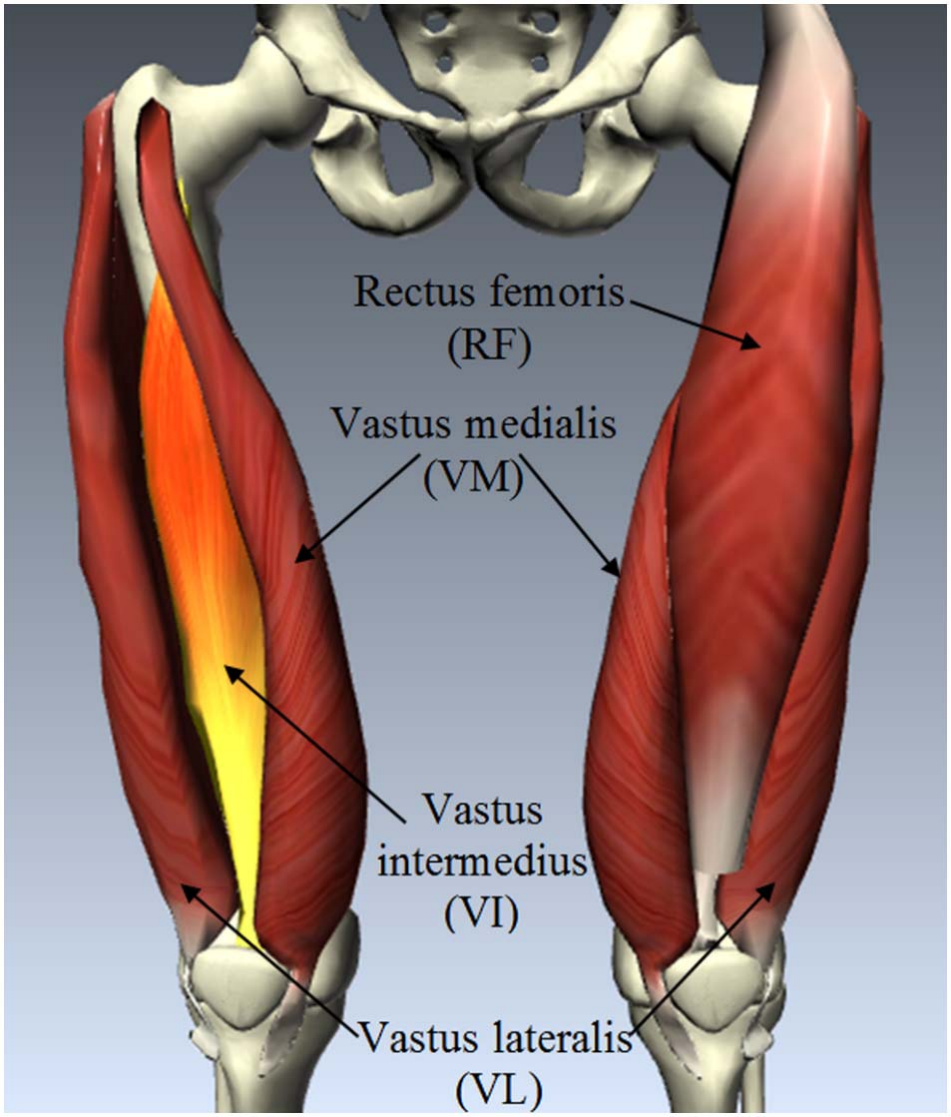
Anatomy of quadriceps femoris muscle group, which includes rectus femoris, vastus lateralis, vastus medialis and vastus intermedius. (Generated using the BioDigital Human Platform, BioDigital Systems, New York City, USA).

To fix this gap, a vibro-ultrasound system was developed in our study. It aims to characterize the skeletal muscle stiffness in the muscle action direction over the entire range of step isometric contraction. This method was then applied on the *vastus intermedius* (VI) to determine the relationship between the VI stiffness and the relative step isometric contraction level of knee extensors, and to evaluate the muscle stiffness difference between the elderly and young female subjects. The VI is one component of the quadriceps femoris muscle group which is shown in Fig. 1. It is crucial to the dynamic stability control for the elderly [2], [3], [44]. It is expected that this novel system can provide us a tool for muscle stiffness measurement under high intensity contraction. The results can help us to better understand the mechanism of sarcopenia, monitor its process on elderly sufferers, and provide useful information during their rehabilitation.

## Materials and Methods

### Ethics Statement

In this study, human subject ethical approval was sought from the Human Ethics Committee of the Hong Kong Polytechnic University, and all the subjects were explained with the experimental protocol and asked to sign on the informed consent form prior to the experiment.

### Vibro-ultrasound System for Muscle Stiffness Measurement

The vibro-ultrasound system consisted of a mechanical vibrator, a programmable ultrasound scanner and a custom-made program for radio-frequency data acquisition. An electromagnetic vibrator (minishaker type 4810, Brüel & Kjær, Nærum, Denmark) which was driven by a power amplifier and controlled by a function generator was used to induce shear waves in the muscle. The vibrator impacted the muscle with a monochromatic low-frequency sinusoidal pulse. In general, the vibration frequency typically adopted in previous studies ranged from 90 to 150 Hz for skeletal muscle stiffness assessment [42], [43]. In this study, 100 Hz frequency was selected to ensure a more reasonable comparison with those studies.

**Figure 2:**
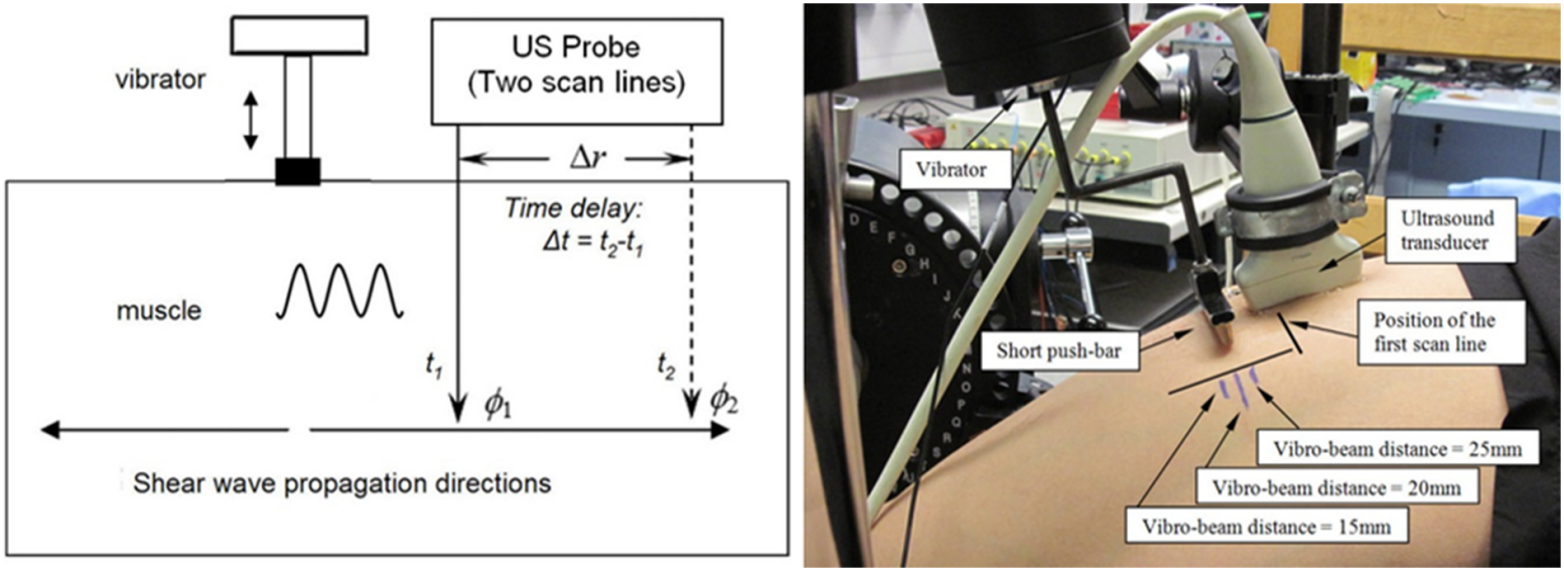
Diagram of the vibro-ultrasound system for shear wave velocity measurement using two ultrasound scan lines (left) and the position of the ultrasound probe and the vibrator during the experiment on one human subject (right). The three positions of the vibrator used for the different vibro-beam distance test were also indicated.



As demonstrated in Fig. 2 (left), shear waves with 10 cycles generated by the external vibrator propagated in the muscle action direction. An ultrasound linear array probe was placed along this direction. At the proximal and distal positions, the tissue movements were monitored by two separated ultrasound scan lines. The distance between the two scan lines was *Δr* (in this study, 15 mm was used) and the time delay between the two detected waveforms was *Δt*. Then the wave velocity *c*_s_ could be calculated by:

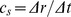
(2)The ultrasound data acquisition system was developed based on a commercial ultrasound scanner SonixRP (Ultrasonix Medical Corp. Vancouver, Canada) with a 5–14 MHz linear array probe (driven by the central frequency 9.5 MHz), and its software developing kits applicable to Visual C++ (Microsoft Corporation, USA). A custom-designed ultrasound transmission and reception sequence was implemented. B-mode images were first acquired (with 256 scan lines corresponding to a 38 mm width) using a predefined penetration depth of 65 mm for helping position the probe at the expected place with a right orientation. For minimizing the anisotropic effects of wave propagation, a straight short push-bar was mounted on the piston of the vibrator and its position was carefully adjusted to guarantee the shear wave would propagate in the muscle action direction [29], [45]. The repetition frequency of the two scan lines for monitoring was finally achieved as 4.6 kHz with the 65 mm penetration depth. Thus the upper limit of shear wave velocity measurement was theoretically 69 m/s, if assuming that the minimal detectable time delay corresponded to 1 frame interval. This value corresponded to a shear modulus of more than 4000 kPa. In addition, the vibrator and the scanner were synchronized by the external trigger. The sampling frequency of the radio-frequency signal was 40 MHz. For each measurement, the subject only needed to maintain the contraction for less than 4 seconds, then 10,000 frames of data were collected and transferred to a computer for further analysis.

The whole experimental setup also included a dynamometer. Isometric torque generated by the knee extensors was assessed using a HUMAC NORM rehabilitation system (Computer Sports Medicine, Inc., Stoughton, MA, USA), which included a specifically designed chair and a fixed dynamometer. The machine was set to the knee joint isolated movement pattern and isometric resistance mode. The knee joint angle can be set and fixed under this mode.

### Data Processing

All radio-frequency signals were processed off-line using a custom-developed program of Matlab (Version R2008, MathWorks, Inc., MA, USA). The main processing steps can be summarized as followings: 1) To arrange the ultrasound signals obtained at the proximal and distal locations into segments; 2) To obtain the transient time shifts of each segment between two consecutive frames; 3) To calculate the displacement waveforms using the transient time shifts and the speed of ultrasound in soft tissue; 4) To detect the peaks of the sinusoidal displacement waveforms; 5) To detect the time delay between each pair of the peaks obtained at the same depth; 6) To calculate the overall time delay between the two waveforms by averaging all the time delay values; 7) To calculate the shear wave velocity using its propagation time and distance.

The tissue displacement waveforms were determined by the normalized cross-correlation algorithm, which is also called the echo-tracking method and widely used in tissue displacement estimation [46], [47]. The method was based on time shift estimation between two congruent segments in pairs of consecutive frames of radio-frequency signals. The time shift was caused by the tissue displacement along the axial direction of ultrasound beam and subsequently the change of travelling distance of the reflected echoes. A segment of reference signal from the initial frame was first defined, and then the most similar segment to this reference one in the subsequent frames was searched using the cross-correlation calculation. The temporal locations of the maximum value of the normalized cross-correlation function marked the time shift between the two segments. Then the tissue displacement was estimated by multiplying the time shift with the velocity of ultrasound. When the time shifts were tracked continuously, a displacement waveform could be correspondingly obtained.

**Figure 3:**
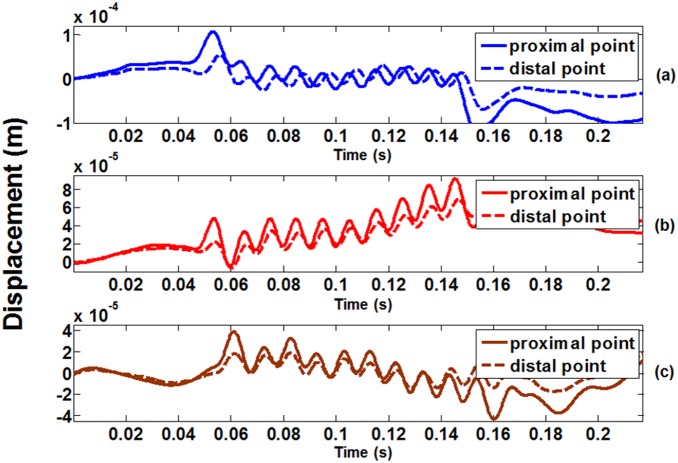
Typical tracking results of tissue displacement waveform of an elderly female human subject. (a) obtained under 0% MVC level (at rest), (b) 50% MVC level, and (c) 100% MVC level. The corresponding shear moduli for (a) to (c) were 9 kPa, 213 kPa, and 574 kPa. The solid line represents the tissue displacement waveforms detected at the proximal location, and the dashed line at the distal location, with reference to the vibration source.

The first frame signal was divided into segments each with 1 mm in depth and with 50% overlap. Each segment was treated as a reference segment and its movement was tracked frame by frame automatically using the cross-correlation method described above. Then the tissue displacement waveforms at certain depths were plotted with time for the distal and proximal locations, as shown in Fig. 3. Fig. 3(a) represents the tissue displacement waveform obtained at 0% MVC level (at rest), Fig. 3(b) at 50% MVC level, and Fig. 3(c) at 100% MVC level (corresponding to shear moduli of 9 kPa, 213 kPa, and 547 kPa). It can be observed that the time delay between the two waveforms became smaller when the contraction level increased, indicating that the shear wave moved faster. To measure this time delay, the peaks of the two displacement waveforms were detected using the zero-crossing points of their first-order derivatives, which were from greater-than-zero values to less-than-zero values. Then the positions of these peaks were plotted and used to determine the time delay values between the two waveforms. Shear wave consists of oscillations occurring perpendicular to the direction along its propagation. Since the shear wave fronts within the ROI were observed as straight lines perpendicular to the time axis, the shear wave propagation direction in VI muscle was confirmed to be perpendicular to the two ultrasound scan lines. That is why the shear wave velocity could be estimated by *Δr* and *Δt* using Eq. 2. Subsequently, Eq. 1 was used to calculate the shear modulus, with the generally used skeletal muscle density of 1000 kg/m^3^ [29].

### Feasibility Tests on Silicone Phantoms

To evaluate the feasibility of the vibro-ultrasound system, shear moduli of several custom-made silicone phantoms with different stiffness were assessed. Their stiffness was compared between the proposed method and the conventional indentation method.

Tissue-mimicking phantoms with a size of 100 mm×80 mm×20 mm were prepared for the experiment. The phantoms were made of addition-curing silicone rubbers RTV-2 (M4600 A/B, Wacker Chemicals Hong Kong Ltd., Hong Kong, China) and their stiffness was varied by adding silicone oil AK-35 (Wacker Chemicals Hong Kong Ltd., Hong Kong, China). The weight ratio between M4600A and AK-35 was selected as 1∶0, 1∶0.25, 1∶0.5, 1∶0.75 and 1∶1, with a decreasing stiffness of corresponding phantoms. The mixtures were then de-aerated in a vacuum cabinet until no more air bubbles were formed due to reduced air pressure. At last, the phantoms were heated at 60°C for several hours to increase the speed of curing. A total of ten phantoms were made (two for each concentration level).

The shear moduli of these phantoms were first assessed using indentation method with a material testing machine (Instron ASTM Method Set, Braintree, MA, USA). The diameter of the indenter *a* is 10 mm. The phantoms were compressed for 2 mm deformation with a rate of 0.5 mm/sec and then relaxed at the same deformation rate. During three cycles of compression-relaxation, the compression load *P* (N) and the deformation *W* (mm) values were collected. Then the Young's modulus *E* was calculated using the Eq. 3, which is based on the Hayes model for the elastic indentation problem of a thin elastic layer bonded to a rigid half-space with a rigid, frictionless cylindrical plane-ended indenter [48].
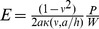
(3)where *h* is the tissue thickness, and *κ* is a scaling factor, which provides a theoretical correction for the finite thickness of the measured phantom and it depends on both the ratio *a/h* and the Poisson's ratio *ν*. The Poisson's ratio *ν* was defined as 0.5 in this study since the silicone phantom is nearly an incompressible material. Then the shear modulus *µ* of the phantoms could be calculated by the following equation:



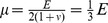
(4)The indentation tests were performed for 3 times on each of the phantoms. Then the stiffness of each phantom was assessed using the vibro-ultrasound system, also for 3 times. To reduce the influence from upper and lower boundaries, the phantom to be measured was placed between two blocks of elastic silicone layers with thickness of approximately 35 mm and shear modulus of approximately 100 kPa. The ROI was 10 mm thick and located in the middle portion of the phantom. All the measurements were performed at room temperature (25±1°C).

The shear moduli were averaged from the 3 measurements and the correlation between the results obtained by two methods were studied statistically. All the data were analyzed using SPSS Statistics (SPSS Inc. Chicago, IL, USA). Statistical significance was set at the 5% probability level.

### Shear Wave Propagation Direction Validation Tests on Young Subjects

It should be confirmed that the shear wave propagates perpendicular to the ultrasound scan lines for the vibro-ultrasound system, so that its velocity can be estimated by a “time-to-flight” method. We assumed that the external disturbance can vibrate VI muscle fibers at different depths simultaneously and the induced shear wave propagates mainly in the muscle action direction. Otherwise, if the shear wave propagates as a spherical wave starting from the vibration source, it would reach the scan line with different propagation time at different depths. Thus when the distance between the two scan lines were fixed, different distance between the vibrator and the first scan line (in short “vibrator-beam distance”) will lead to the different value of the measured shear wave velocity. Accordingly, the experiment was designed to verify whether the vibrator-beam distance would affect the measured shear wave velocity on human subjects.

Since this experiment was just to verify the feasibility of our approach, elderly subjects were not recruited this time. Ten healthy young subjects (8 males and 2 females, age: 30.7±4.1 yr, height: 170.2±10.7 cm, weight: 68.0±14.4 kg) were included in this experiment. The subject was asked to sit on the chair with several straps restraining his/her waist and shoulders. A cuff was fastened around the right lower leg and fixed to the lever of the dynamometer. The axis of the lever was aligned with the supposed rotation axis of the right knee joint. With the guidance of B-mode images, the vibrator and the ultrasound probe were hung right above the middle part of the RF muscle belly with a predefined distance (if this distance was 10 mm, the vibrator-beam distance was approximately 20 mm), as shown in Fig. 2 (right). Ultrasound gel was applied between the ultrasound probe and the skin. The ultrasound probe was first adjusted to be aligned in the direction along the muscle fibers under the guidance of B-mode images (The muscle fibers could be seen as parallel straight lines in the images. If the ultrasound plane was at an angle to the fibers, the fibers would not be shown as clear lines). Then the straight short push-bar was adjusted to be perpendicular to the probe and muscle fibers also under the guidance of ultrasound images. Both the push-bar and the probe were then rigidly fixed. With the help of this push-bar, planar pattern shear wave would be generated at the expected depth and propagate in the direction of muscle action [40].

The experiment was performed at a 90° knee joint angle. First, the MVC torque was assessed as the highest torque value produced from three successive isometric contractions which were maintained for 5 seconds with about 30 seconds interval for rest. Next, the muscle stiffness was assessed at relaxed condition and at 20% MVC level, with three different vibrator-beam distances, i.e. 15 mm, 20 mm and 25 mm. For each measurement, the subject was asked to maintain the isometric contraction for approximately 4 seconds, and three repeated measurements were made under the same condition with 1 minute interval for rest.

The shear modulus was represented by the mean value of the three repeated measurements. The shear moduli of 10 subjects measured at different vibrator-beam distances were plotted using a linear correlation model, and then were analyzed using two-way repeated measure analyses of variance (ANOVA) to evaluate their differences. The normalized root mean squared deviation (NRMSD) among the results measured at different vibrator-beam distances was also calculated. The definition of NRMSD is as follows:



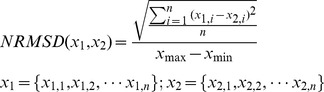
(5)where *x*_max_ and *x*_min_ are the maximal and minimal values among the observed results. NRMSD value is often expressed as a percentage, where lower values indicate less residual variance.

### Muscle Stiffness Measurements on Elderly and Young Female Subjects

Ten healthy elderly female subjects (age: 56.7±4.9 yr, height: 156.9±5.6 cm, weight: 58.9±8.4 kg) and ten healthy young female subjects (age: 27.6±5.0 yr, height: 164.3±4.4 cm, weight: 55.3±4.0 kg) volunteered to participate in this part of the study. The experimental setup was almost the same as described in the above section, and all the recruited subjects were also explained with the experimental protocol and asked to sign on the informed consent form prior to the experiment. However, to make the elderly subjects more comfortable, the experiment was performed at a 60° knee joint angle, but not 90°. The distance between the vibrator and the probe was set to be approximately 10 mm. The MVC torque value was also assessed first. Next, the muscle stiffness was measured for three times at relaxed condition. Then the subject was asked to maintain isometric contraction at different MVC levels, from 10% to 100%, with an increase of 10% for each step. At each MVC level, three assessments were performed with about 1 minute interval for rest. The shear modulus of each individual subject was represented by the mean value obtained from the three repeated measurements. Therefore, a total of 660 (20 [subjects] ×11 [contraction levels: 0%–100% MVC] ×3 [three times]) measurements of VI shear modulus were performed by the same investigator. The intra-class correlation coefficient (ICC) was used to evaluate the intra-observer repeatability. The shear moduli measured at the same contraction level across the ten elderly subjects and ten young subjects were then averaged and used to investigate the relationship between muscle stiffness and relative isometric contraction levels (% MVC). To determine the pattern of this relationship, polynomial regression analyses by linear, quadratic and cubic models were performed for each individual, and the coefficients of determination (R^2^) values of these models were compared using paired samples T-test. Since we found that the quadratic regression model had the best performance, the mean shear moduli across the ten subjects were then fitted with the relative isometric contraction levels (% MVC) using a quadratic regression model. To study the difference of the VI stiffness between elderly and young female subjects in a relaxed state and at different isometric contraction levels, two-way repeated measure analyses of variance (ANOVA) (Age [young and elder] × % MVC [0%–100%, 11 levels]) were used to analyze the measured shear modulus. Specially, the comparison of the VI shear modulus measured in a relaxed state (0% MVC) was first performed separately using one-way ANOVA method.

## Results

### Feasibility Tests on Silicone Phantoms

**Figure 4:**
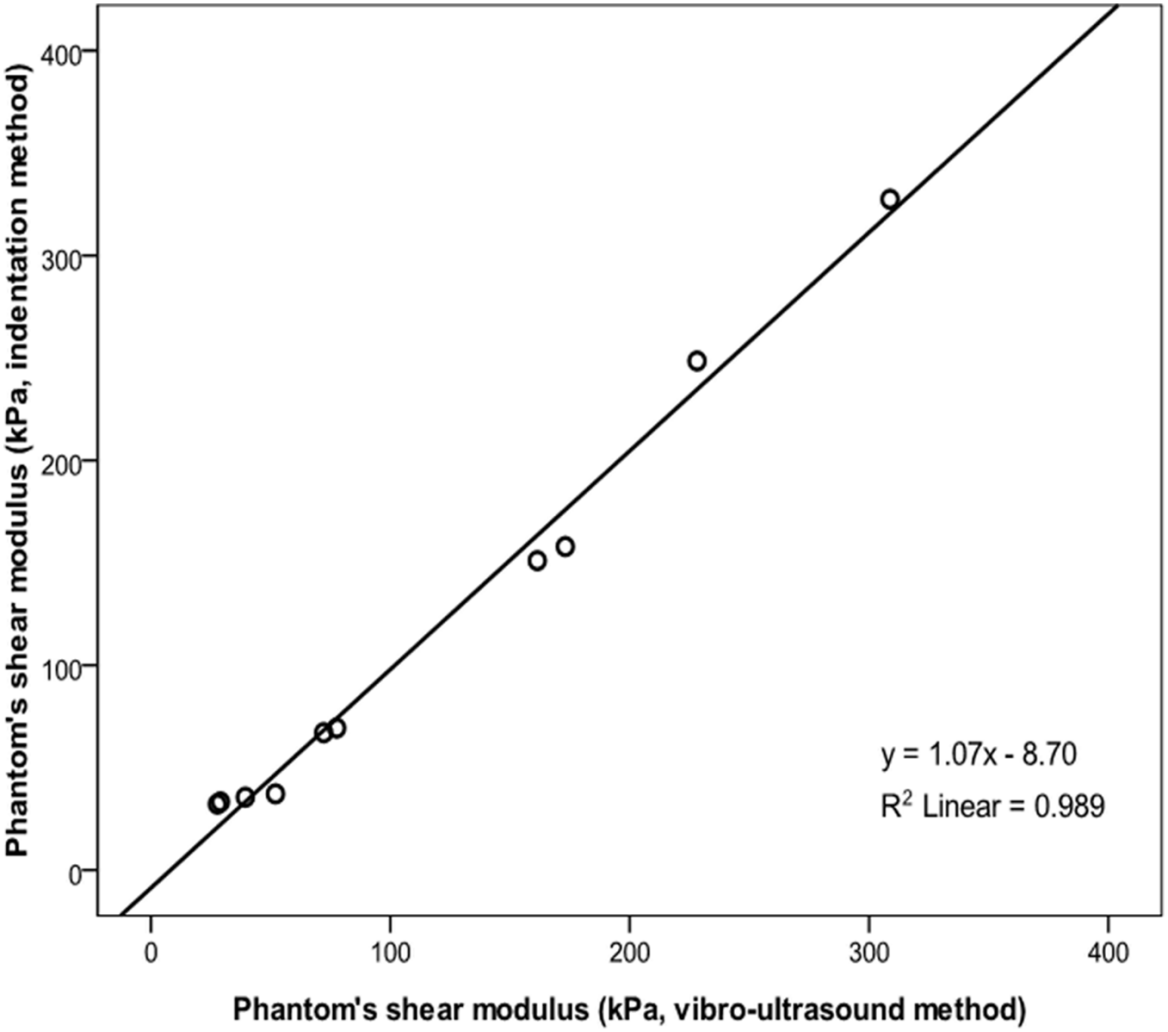
Correlation between the shear modulus values assessed by indentation method and the corresponding values measured by the vibro-ultrasound method.

A good linear correlation was found between the results obtained by the indentation method and those measured using vibro-ultrasound system for the 10 silicone phantoms, with a regression of *y = 1.07x−8.70* and R^2^ value of 0.989, as shown in Fig. 4. This demonstrated that the vibro-ultrasound system was feasible for measuring the stiffness of tissue-mimicking phantoms and could be used for monitoring the change of muscle stiffness in a large measurement range.

### Shear Wave Propagation Direction Validation Tests on Young Subjects

**Figure 5:**
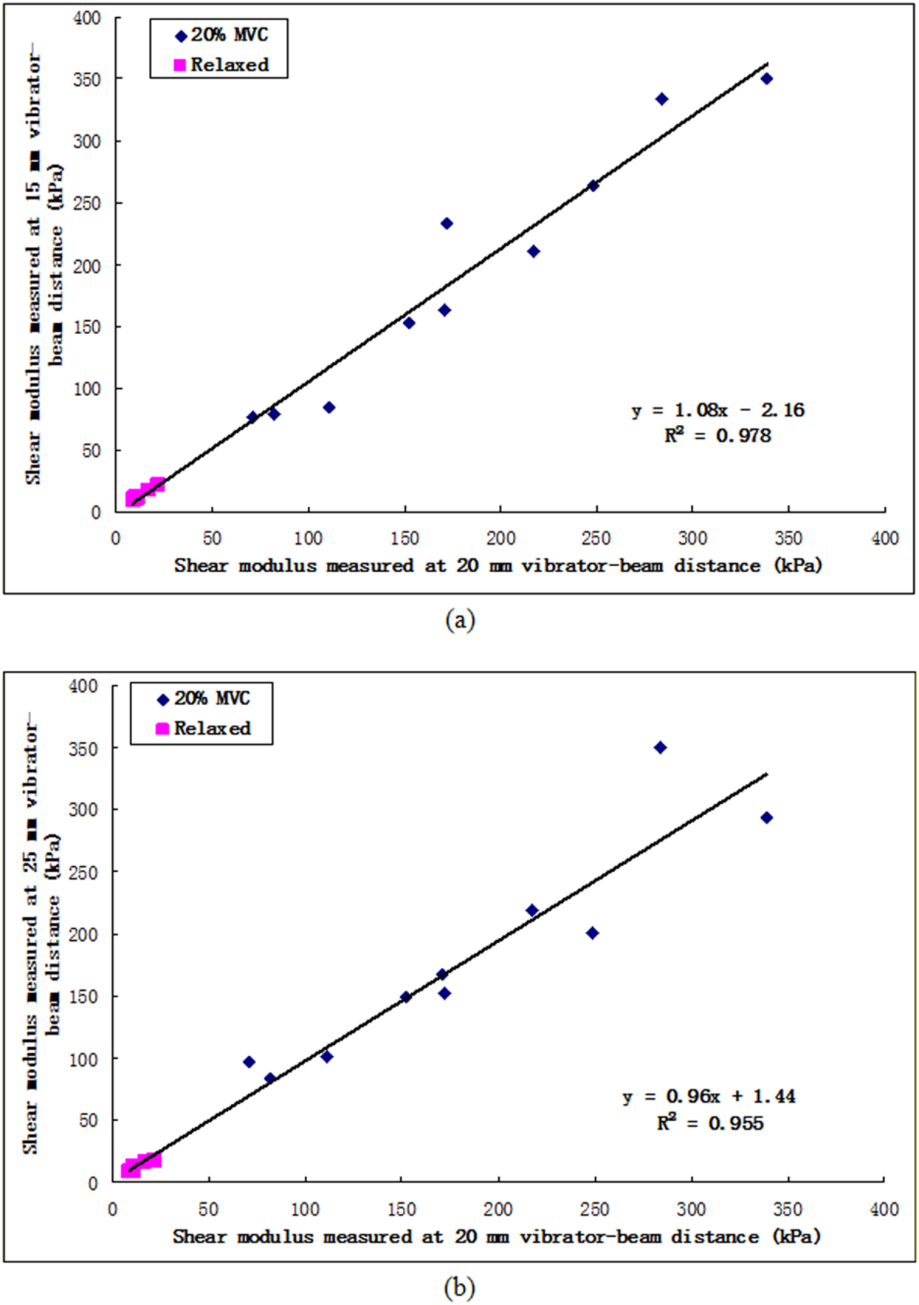
Comparison of the shear moduli between (a) 15 mm and 20 mm; and (b) 25 mm and 20 mm, revealed that the vibrator-beam distance appeared to affect very little on the measurement result of shear modulus.

Fig. 5 shows the comparison of the shear moduli of VI measured at different vibrator-beam distances. The R^2^ value was 0.978 and 0.955 for the correlations between the results measured at 15 mm and 20 mm vibrator-beam distances and between those at 25 mm and 20 mm, respectively. There was no significant individual effect of vibrator-beam distance (p = 0.818). On the other hand, the individual effect of isometric contraction levels was significant (p<0.001). The NRMSD values were 5.6% and 6.5%, respectively. The results demonstrated that the measured muscle shear modulus would not be significantly influenced by the small variation of vibrator-beam distance. It also provided another evidence to support the assumption that in this study the pattern of shear wave was quite close to planar when it propagated in the muscle action direction of VI.

### Muscle Stiffness Measurements on Elderly and Young Female Subjects

**Figure 6:**
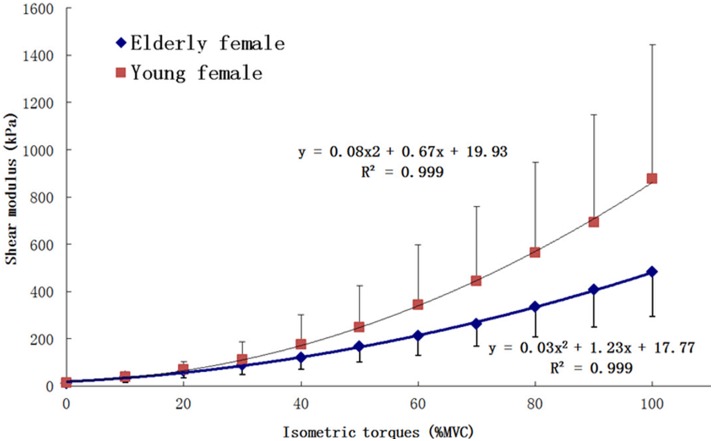
The averaged VI shear modulus of ten elderly healthy female subjects and that of ten young healthy female subjects, plotted with the different relative isometric contraction levels (% MVC torque). The error bar represents the standard deviation among the 10 subjects.

The overall ICC for the measured shear moduli on all female subjects was 0.994, suggesting a high degree of reproducibility of the measurements. The mean R^2^ values of the polynomial regression analyses by linear, quadratic and cubic models on the relationship of shear modulus vs. % MVC level were 0.932±0.034, 0.995±0.004 and 0.997±0.003, respectively. The results of paired samples T-test indicated that there was no significant difference between the R^2^ values of quadratic model and cubic model (p = 0.153). However, significant differences were found between the R^2^ values of linear model and the other two models (both p<0.001). Thus the quadratic regression model was selected to correlate the mean shear moduli with the relative isometric contraction levels, as shown in Fig. 6. The result indicated that the VI stiffness of both elderly and young female subjects in the muscle action direction was positively correlated to the relative muscle activity intensity (% MVC) of the knee extensors over the entire range of step isometric contraction. The mean VI shear modulus of elderly and young female subjects in a relaxed state (0% MVC) was 12.8±5.4 kPa and 9.5±3.3 kPa, respectively. And results of the one-way ANOVA showed that for “Age” factor there was no significant effect on VI shear modulus (p = 0.106) in a relaxed state. For VI shear modulus measured under different step isometric contraction levels, results of the two-way ANOVA showed that the main effects for “Age” and “% MVC” factors on VI shear modulus were both significant. The estimated marginal mean value of VI shear modulus of the young female subjects was larger than that of the elderly participants (p<0.001). Furthermore, the two-way interactions of the two factors were also significant (p = 0.01). With the increasing of “% MVC”, differences between the VI shear modulus of young female subjects and that of elderly subjects also increased, which was also directly indicated in Fig. 6.

## Discussion

### Vibro-ultrasound Muscle Stiffness Measurement System

In this study, a vibro-ultrasound system was developed and applied for muscle stiffness assessment on the VI muscle of elderly female subjects. The good agreement between the results obtained on silicone phantoms by both our system and the conventional indentation method suggested that the system was feasible for tissue stiffness assessment in a large measurement range. Moreover, the high ICC value (0.994) of the measured shear moduli of VI muscle indicated that the measurement was highly repeatable.

The shear wave velocity was determined by the distance and the propagation time between the two ultrasound scan lines in our system. The relatively larger distance (15 mm) and higher frame rate (4600 frames/second) help to achieve a better measurement range of shear modulus comparing to the existing methods. In addition, since a mechanical vibrator was used, the amplitude of shear wave generated by our system (50–80 µm at the proximal scan line, and 20–50 µm at the distal scan line) was much larger than the method based on acoustic radiation force (generally less than 10 µm) [24]. Therefore, the effect of jitters on the displacement waveforms of our system would be much smaller in comparison with the acoustic radiation force based methods, and this would also help to improve the precision of our measurements.

However, there is still some uncertainty on the accuracy of the presented measurement results. On self-made phantoms, we only confirmed the accuracy of this system with the shear modulus up to about 350 kPa (for the hardest one, the measured shear modulus was 347.2±22.3 kPa with our system and 327.5±6.4 kPa with indentation method). To our knowledge, this value is 30% larger than the maximal measurement upper limit of the existing methods, i.e. 266 kPa for SSI system [37], but is still smaller compared to the reported results measured on the elderly and young female subjects in this study (under 100% MVC level, the mean shear modulus of VI among ten elderly female subjects was 482.5±189.6 kPa, the minimal and maximal values of individual were 148.1±27.6 kPa and 1114.5±151.4 kPa, respectively; and the mean shear modulus of VI among ten young female subjects was 877.5±566.7 kPa, the minimal and maximal values of individual were 525.7±56.9 kPa and 2454.9±260.6 kPa, respectively). Although these results are theoretically credible, it is still uncertain whether they are equally accurate, since they are beyond the confirmed measurement range and the stiffness of silicone phantom can be hardly increased (for the hardest phantom, we did not add any silicone oil to soften it). Future studies should confirm this by finding other materials which can be used to make the phantom with higher stiffness.

In current method, a simple biomechanical model (Eq. 1) was adopted to calculate the shear modulus of tissue, assuming the tissue was pure elastic. While in some studies using SDUV and SSI, a more complicated model, Voigt model, has been used to estimate the shear modulus and shear viscosity simultaneously, based on the frequency-velocity dispersion curve. Some previous studies by Gennisson et al. and Deffieux et al. have reported that the shear wave velocity remained almost constant in the muscle action direction when the exciting frequency changed under both relax and isometric contraction conditions of *in vivo* muscle [31], [49]. On the contrary, Chen et al. and Urban et al. found the velocity was frequency dependent [24], [32]. The difference may be caused by their experimental designs. From another aspect, Gennisson and Deffieux conducted the measurement on human muscles *in vivo*, while Chen and Urban tested bovine and porcine muscles *in vitro*. In Chen’s study, the bovine muscle was punched with a through hole at its center and a glass rod was glued in the hole throughout the thickness of the sample. Thus the structure of muscle fibers was destructed and its mechanical properties should be different with that *in vivo*. In addition, it was shown in Urban’s study that the shear modulus measured across muscle fibers (in transverse direction) was dependent on shear wave frequency, but that measured along muscle fibers (in the muscle action direction) was almost constant with the frequency increasing. This finding was consistent with those reported by Gennisson and Deffieux. Accordingly, the viscous effect can be neglected when a skeletal muscle is tested *in vivo* for its elasticity in the muscle action direction using shear wave propagation methods. Such a conclusion needs further verifying for different muscle groups under different physiological and pathological conditions.

The wave propagation pattern in skeletal muscle is another important factor which would affect the results and should be carefully studied and controlled. In our system, shear wave velocity was estimated by dividing the distance between the two scan lines with the corresponding wave propagating time. This required the wave front to be parallel to the two scan lines. If the wave front has a spherical or oblique plane surface, the distance used for calculation would theoretically be longer than the real length of the wave travelling path, resulting in overestimated shear modulus. To verify this issue, Sack et al. reconstructed the shear wave pattern under different conditions using *in vivo* MRE data collected on biceps brachii muscle [40]. In their results, planar wave pattern was observed when the external excitation was applied on muscle belly using a short push-bar, of which the orientation was kept to be vertical to the muscle fibers to minimize the anisotropic effects. In our study, the similar setup was used to facilitate the wave pattern be close to planar. Furthermore, the measured muscle shear modulus has been proved to be not significantly influenced by the small variation of vibrator-beam distance, which provided another evidence to support the assumption of planar wave that propagating in the muscle action direction.

In this study, a mechanical vibrator was applied to generate shear waves in a deep muscle, VI, with enough amplitude and hence increase the SNR of the detected tissue displacements. Mechanical vibrator was generally considered to cause more wave reflection at the interface of different tissue layers, especially when there is complex bone-muscle geometry [50]. However, the VI has a large size, so plane shape and its muscle fibers are almost aligned along the same axis of muscle action direction (the pennation angle is less than 10°) [51], the reflection phenomenon in wave propagation direction would be very small and not affect the results. In our pilot study, it was observed that the skin surface stress imposed by either the mechanical vibrator or ultrasound transducer would also influence the wave pattern by damping the vibration of underlying tissue. As a deep muscle like VI, such effect could be mostly avoided by fixing the vibrator and transducer rigidly to restrict the relative motion to the muscle.

### VI Stiffness of Elderly and Young Female Subjects in Relaxed Condition

In relaxed condition, the measured mean shear modulus of VI in ten elderly female subjects was 9.5±3.3 kPa and in ten young female subjects was 12.8±5.4 kPa. Although several methods have been used to estimate the shear modulus of skeletal muscle, few studies have been performed on elderly female subjects and even more few on the VI muscle. Domire et al. measured the stiffness of tibialis anterior muscle at a relaxed condition on 20 female subjects with an age range of 50 to 70 years using MRE [43]. They reported that there was no significant effect of age on the stiffness of relaxed muscle, which is in agreement with ours. Kot et al. measured the shear modulus of RF in relaxed condition on young healthy subjects (14 males and 6 females, mean age 26.4±3.5 yr) and the mean value was 12.78±3.56 kPa, which is almost in the same range of ours [52]. Bensamoun et al. measured the shear modulus of VL and *vastus medialis* (VM) in relaxed condition on young healthy subjects (4 males and 10 females, mean age 25.2±1.78) [38]. The mean values were 3.73±0.85 kPa and 3.91±1.15 kPa, which is smaller than ours. Besides the effects for different age ranges, the different stiffness observed in different muscles may also indicate that the propagation of shear waves is influenced by the muscle structure, such as muscle fiber orientation. The RF and VI, which are bipennate muscles, exhibit higher muscle stiffness than the VL and VM, in which the fiber orientation is unipennate [38]. In addition, these differences may be also related to the muscle volume, fiber type distribution, function or other specific characteristics of different muscles. Although it is difficult to compare these results directly, all these shear modulus values in relaxed condition fall into a similar range.

The results in relaxed condition may be also influenced by other factors. All the subjects were asked to fully relax their muscles during the tests, but they might have slightly contracted their muscles unconsciously. Since muscle stiffness is strongly influenced by the muscle contraction, this kind of slight tension might affect the measurement results. In addition, the momentary muscle stiffness might also change with their preceding usage before the measurements [41]. This was the reason that the subjects were asked not to participate in any strength or flexibility training one day before the measurement in our study.

### Age Effect on VI Stiffness under Different Step Isometric Contraction Levels

Our results demonstrated that the stiffness of VI muscle in the muscle action direction was positively correlated to the relative muscle contraction level (% MVC torque) over the entire range of step isometric contraction for both elderly and young female subjects. Some previous studies have used different weight loads imposed on the muscle to represent different contraction levels, such as on the knee extensors [28] and on the elbow flexors [39]. Their results have also reported that the muscle stiffness was positively correlated to the increasing weight loads. However, using weight load as an indicator of isometric contraction intensity was not accurate enough, since the muscle strength was different among the subjects and the lever arm of force was not counted. In other studies, the % MVC torque has been used to represent the muscle activity intensity level. However, in these measurements, the muscle contraction ranges rarely reached 50% MVC torque level due to the upper measurement limitation of the methods they used. For example, on young healthy subjects, Bensamoun et al. measured the shear modulus of VL and VM at 10% and 20% MVC torque, and it was 6.11±1.15 kPa and 8.49±4.02 kPa for VL, while it was 4.83±1.68 kPa and 6.40±1.79 kPa for VM [38]. They concluded that the shear modulus of VL and VM both increased significantly with the increase of % MVC torque. Although it was difficult to compare the shear modulus they measured with ours due to the different muscles and different age ranges, our conclusion that the muscle stiffness increased with the increasing isometric contraction level was in good agreement with their finding.

In this study, it was also found that the relationship between the muscle stiffness and relative isometric contraction level could be better represented using a quadratic curve over the entire range of isometric contraction. This relationship was mostly reported to be linear in previous studies [28], [39]. In their studies, the shear modulus was only measured under 3 or 5 different isometric contraction levels at low intensity range, which may explain why different correlation was observed in our study, since a small portion of the quadratic curve can be treated as being linear.

We also noticed that the standard deviations of the mean VI shear modulus values increased substantially with % MVC levels. We thought this mainly came from the differences of the individual muscle strength of the subjects. At lower % MVC levels, these differences were not obvious, and thus the differences of muscle stiffness among the subjects were also small. However, when performing high intensity contraction, the differences of the absolute torque values corresponding to the high % MVC levels became larger due to the individually different MVC torque.

We found that the mean VI shear modulus of the young female subjects was larger than that of the elderly participants, especially at the relatively higher step isometric contraction levels. Rare previous study has been reported on the age difference of muscle shear modulus under different step isometric contraction levels. Ochala et al. [31] measured the musculotendinous stiffness of plantar flexors at 20%, 40%, 60% and 80% MVC levels on young and elderly subjects. The results indicated that the musculotendinous stiffness of elderly subjects was smaller than that of young subjects measured at the same contraction level. Although musculotendinous stiffness can only reflect the global mechanical properties of musculotendinous complex, their results are still a valuable reference for ours. Further studies should be performed to reveal the internal relationship between the muscle morphology changes, the muscle fiber atrophy and the decreasing muscle stiffness in its aging process.

Although for the first time muscle stiffness over the entire range of isometric contraction was measured with a further verification of the correlation between muscle stiffness and % MVC torque, there are still some limitations in our study. The measurement range of current vibro-ultrasound system should be further improved for young and male subjects by increasing the frame rate of the ultrasound acquisition, and by accurately controlling the position, frequency and amplitude of the shear wave generated in the skeletal muscle. The gender, age and joint angle dependences of the muscle stiffness under different isometric contraction levels should be also studied. The pennation angle of VI muscle tested in this study is less than 10° [51] and roughly equal to the muscle action direction, so it is relatively easy to align the shear wave propagation direction along the muscle fiber direction. However, the pennation angles of other muscles may be very different. Further study is required to test the performance of the proposed system for the muscles with large pennation angle, so other skeletal muscles can be also studied by the developed vibro-ultrasound system.

Our aim is to provide real-time muscle stiffness measurement simultaneously with some other signals, such as sonomyography (SMG) which are the sonographically detected signals of the architectural change of muscles [53], B mode ultrasound images, force or torque values, joint angle and surface EMG. Combining these signals into one system will help us further understand the function of skeletal muscles.

## Conclusions

In this study, a vibro-ultrasound system was developed for skeletal muscle stiffness measurement. Feasibility test on silicone phantoms demonstrated that this system was capable of measuring the shear modulus of tissues. Then, this system was applied for *in vivo* tests on elderly and young female human subjects. For the first time, the relationship between the muscle stiffness of VI and the relative step isometric contraction level (% MVC torque) was studied over the entire range, i.e. from 0% to 100% MVC. A quadratic polynomial curve was found to well represent the correlation between the two parameters. These results provided additional information about the recruitment strategies of muscles under step isometric contraction, which needs to be further investigated. In addition, it has been shown that there was no significant difference between the mean VI shear modulus of the elderly and young female subjects in a relaxed state (p>0.1). However, when performing step isometric contraction, the VI stiffness of young female subjects was found to be larger than that of elderly participants (p<0.001), especially at the high contraction levels. The vibro-ultrasound system could be further improved and combined with other signals to provide better understanding of skeletal muscles.
